# Revisiting the Response to the 1977 Book *The Myth of God Incarnate*

**DOI:** 10.1177/00033286251316169

**Published:** 2025-02-06

**Authors:** Robert Revington

**Affiliations:** Queen’s University, Canada

**Keywords:** Church of England, Dennis Nineham, Don Cupitt, Incarnation, John Hick, Maurice Wiles, Myth of God Incarnate, twentieth-century theology

## Abstract

In 1977, SCM Press published a controversial theological book titled *The Myth of God Incarnate*. Although the authors had close ties to the Church of England, their book denied the divinity of Christ. It caused an uproar. This study revisits contemporary responses to the book and examines why so many of them were negative. The book was viewed as a failure by many observers because it was supposed to be a work of “popular” theology—though written by serious scholars—but many reviewers found it both inaccessible for a popular audience and too superficial for an academic one. Furthermore, it was publicized in a way that was insensitive to the faith of churchgoers. Finally, the book’s approach is a prime example of what Peter L. Berger called “the self-liquidation of the theological enterprise.” In short, if the book’s conclusions were accepted by the Church, they would undermine the church’s existence.

In 1977, SCM Press released a controversial theological book titled *The Myth of God Incarnate*.^1^ The contributors (Don Cupitt, Michael Goulder, John Hick, Leslie Houlden, Dennis Nineham, Maurice Wiles, and Frances Young) taught at the universities of Oxford, Cambridge, and Birmingham, as well as the Anglican seminary Ripon College. Although the authors had close ties to the Church of England, their book denied traditional orthodox Christian ideas about the divinity of Christ. As a result, the book caused an uproar. As the back cover of its fourth printing recounts, the book’s “seven authors were described as ‘Seven against Christ’ and there was an attempt in the court to defrock those of them who were Anglican clergymen.” The phrasing of “Seven Against Christ” reuses the common phrasing used for the authors of the 1860 theological book *Essays and Reviews*—which also featured controversial essays by prominent figures in the Church.^2^ Ieuan Ellis describes the controversy surrounding that earlier book as follows:

To examine the periodicals, books and pamphlets also becomes an exercise in the pathology of Victorian religious thought which has nothing to equal it. Amid the hysteria and fear of crisis sensible men lost their heads, and sarcastic writers preferred to use invective.^3^

*The Myth of God Incarnate* may not have provoked *as great* a reaction, but there are striking similarities. Both books were essay collections featuring contributions by Church of England clergymen that espoused controversial theological ideas. Regardless, despite the fact that reactions to *The Myth of God Incarnate* were “violent,” the back cover adds that “the first impression was sold out on publication day and a substantial reprint followed soon afterwards.” Indeed, within eight months, the book sold thirty thousand copies, even as the Church of England Evangelical Council requested that “the five Anglican authors . . . resign their orders.”^4^ Even so, the reception of the book is multifaceted.

When one looks at contemporary reviews of *The Myth of God Incarnate*, it is striking that they are almost all negative. One of the common threads is that *The Myth of God Incarnate* failed by not working adequately at either pole by which it could be judged. It was supposed to be a work of popular theology that laypeople could read, though written by serious scholars. Yet the book is neither accessible for a popular audience, nor was it seen as a substantial work of scholarship by academic reviewers. Not only is the book too dense for lay audiences, but everything about its presentation—from its controversial title to its publicity—appeared deliberately provocative, insensitive, and even incendiary to the faith of the churchgoers who were supposed to read it. A theological publication that holds a press conference openly denying the divinity of Jesus is always going to upset people. In short, I argue that *The Myth of God Incarnate* was received so negatively because the book was written in a gray area that made it insufficient both as a popular book for the churches or as a work of serious scholarship. Many of the academic reviews are savagely critical. Furthermore, it was negatively received by Christians because it participates in what Peter L. Berger called “the self-liquidation of the theological enterprise.”^5^ In other words, following Berger’s analysis of certain theological trends, the problem with *The Myth of God Incarnate* was that—despite the authors’ protests to the contrary—if their conclusions were accepted by the church at large, they would ultimately undermine the church’s existence.

## The book and its response

The book’s core ideas threaten the foundations of the Church. To that end, a brief summary is in order. The preface emphasizes that Christianity must continue to change, that “modern scholarship” proves that there is no unifying set of beliefs in Christian history, and that “‘orthodoxy’ is a mirage.”^6^ The first essay by Maurice Wiles concludes that the traditional understanding of the Incarnation must be abandoned but that such language “would still seem appropriate as a pictorial way of expressing these truths.”^7^ Frances Young argues that the Church’s Christology developed gradually; she concludes that “the future seems to lie with pluralism in Christology.”^8^ Michael Goulder’s essays also deny the Incarnation and then Goulder examines two Christian “myths” that he considers foundational to Christian origins: a “Galilean eschatological myth” and an alleged Gnostic myth from Samaria.^9^ Another essay by Young speculates further on how the Church’s Christology might have developed—with Greco-Roman influence—to exalt Jesus as God.^10^ Later, Maurice Wiles further probes the meaning of “myth” as it relates to traditional Christian doctrine.^11^ John Hick compares Christianity to other world religions and expresses his hope that Christians will become more pluralistic and “outgrow” a literal understanding of the Incarnation.^12^ The cumulative aim of the essays in the book is to argue that Jesus was not God and to attempt to explain how only *later* Christians came to see him as God. As such, the book’s conclusions would theoretically undermine belief in much of the Apostles’ Creed—even as the authors simultaneously try to maintain that the Church can somehow exist *without* a belief in the Incarnation.

Not only did the book make the claims it did, but it was marketed in a provocative way. One person who reflected on this point was the New Testament scholar F. F. Bruce. Bruce was a professor at the University of Manchester who was from the Open Brethren tradition (an offshoot of the conservative Plymouth Brethren). Even though he was a conservative Christian New Testament scholar, Bruce wrote that, if *The Myth of God Incarnate* had had a more innocuous title such as “*Ten Essays in Christology*, it would not have created the stir it did.”^13^ Bruce claims that “the inclusion of the emotive word ‘myth’ in the title was more responsible for the stir than the contents of the book.”^14^ While Bruce thinks that a less provocative title would have caused less controversy, it is hard to dispute the fact that the title accurately reflects the contents of the book. Likewise, the theologian David L. Edwards described the title as “inflammatory” because, in his words, “to call a book *The Myth of God Incarnate* is as provocative as it would be to call a book this jubilee summer *The Myth of the Queen’s Reign*.”^15^ Edwards acknowledges that “the contributors could go on to say that the Queen reigns in some sense and is good at it, but still they would have provoked.”^16^ At the time the book was published, Edwards was a canon at Westminster Abbey and the Chaplain to the Speaker of the House of Commons.^17^ In contrast with Bruce, Edwards’s analogy accurately reflects the intentions of the book’s creators. John A. T. Robinson also called the title “most unfortunate” because of the associations that the word “myth” would create.^18^ Indeed, it is hard to avoid the conclusion that the authors and publishers *wanted* to cause a stir. As has been alluded to, on June 28, 1977, in a headline-grabbing press conference for the book, the book’s editor (and contributor) John Hick explicitly denied that Jesus ever thought he was God but did call him “the most wonderful human being who ever lived.”^19^ When discussing this press conference, John F. Johnson wrote: “Perhaps no other theological work of recent times has been so popularly heralded.”^20^ By having a press conference—for a book with that title—surely Hick knew what would happen. Robinson says that the book should never have been promoted “with a press-conference” and reflects that the authors must have known that they picked a title “bound to produce more heat than light.”^21^ Robinson maintains that the book’s title (and the title of individual chapters such as “Christianity Without Incarnation?”) was always going to alienate people.^22^ It should be noted that Robinson’s personal experiences may have influenced his conclusions here. Robinson had first-hand experience about how an incendiary title could affect the reception of a piece of theological writing. Although he was the Dean of Chapel at Trinity College, Cambridge by the time he wrote this review, Robinson was most famous for his controversial 1963 book *Honest to God*—written while he was the Bishop of Woolwich.^23^ His experiences from the uproar caused by *Honest to God* seem to have influenced his critique of *The Myth of God Incarnate.* Robinson believed that much of the controversy for his own book could be traced to a newspaper article he published in the *Observer* in March 1963 before the book itself was published.^24^ The article was given the provocative title “Our Image of God Must Go,” and when he reflected on it later, Robinson recalled:

Much of the hurt was caused by the title, which was not of my choosing, though I accept full responsibility. My original title, “A New Mutation in Christianity?,” was designed to be positive and to draw attention to the contribution of Dietrich Bonhoeffer, from which I began and on which I intended the emphasis to fall . . . The one suggested to me—“Our image of God must go”—struck me as negative and arrogant. I resisted it, but under pressure of time and with nothing convincing to propose in its place, I eventually concurred. Journalistically it was a good title, as events were to show. But as well as leaving a destructive impression, it also had the effect of shifting the centre of gravity of the subsequent debate.^25^

It is striking to read those words from his own experience alongside his criticism of the use of the word *Myth* in the provocative title to Hick’s book. Still, he was not the only one to criticize the title *The Myth of God Incarnate*.

To that end, one of the most common criticisms of the book was that it was imprecise in its understanding of the meaning of the word “Myth”—which just so happened to be the most controversial word in its title. Herbert McCabe—a Dominican priest at Blackfriars Priory in Oxford—criticized the authors for being unclear in explaining whether a “myth” was the same thing as an “untruth.”^26^ Similarly, David Willis—a scholar who taught at the San Francisco Theological Seminary and Princeton—criticizes the book for not clearly defining “myth” in relation to other types of truth.^27^ Likewise, although Rowan Williams later became the Archbishop of Canterbury, he wrote a similar review of the book early in his career, around the time he began to teach theology at Cambridge. Williams’s review argued that “myth” was not equivalent to falsehood but had value as a means of conceptualizing religious truths.^28^ Williams concluded that “incarnational dogma in its classic form does serve as a life-giving myth, a map of reconciliation.”^29^ Similarly, Robinson affirmed that “myth is a profound form of truth, and not the opposite of truth as the deliberate antithesis of the titles *The Myth of God Incarnate* and *The Truth of God Incarnate* would suggest.”^30^ Granted, Robinson is also critical of the rebuttal volume *The Truth of God Incarnate* for being rushed and disorganized and for not doing justice to the meaning of “myth” either.^31^ Conversely, Alasdair Heron—a Scottish theologian then teaching at the Irish School of Ecumenics at Trinity College, Dublin—writes that *The Truth of God Incarnate* does a better job of explaining the significance of incarnation than *Myth*—and that people should buy the former book but could keep their money rather than purchase the latter.^32^ Johnson also rips *The Myth of God Incarnate* for the authors’ “inconsistent view of what constitutes a myth.”^33^ James E. Griffiss—an American Episcopal theologian who taught at Nashotah House in Wisconsin—described *The Myth of God Incarnate* as “untidy” because “it did not make clear in what sense the word ‘myth’ was used” and “did not make clear in what sense ‘God Incarnate’ was used.”^34^ Jerry H. Gill and John Stott had similar criticisms.^35^ In *The Expository Times*, Cyril S. Rodd concluded that the authors used the word “partly, it seems, because it is provocative.”^36^ Rodd adds that this “is a spurious provocativeness,” but “we regret that the writers should have felt constrained to use such a slippery word which cannot assist the exploration of serious religious issues.”^37^ Rodd was a Methodist theologian who taught at the Roehampton Institute of Higher Education in London. At any rate, it is overwhelming to see how many reviewers—of different persuasions—accurately seized on this fundamental problem: that the book’s contributors should have been clearer in the way they used the word “myth,” or recognized that it was an oversimplification to merely equate the word with falsehood.

In any event, soon after the book was published, Desmond Albrow wrote in the *Sunday Telegraph* that for most Christians, “it is still faith and not theology that makes them whole,” but that their faith would “survive most of the semantic evasions” from the book’s press conference.^38^ Albrow also reacted with sarcasm to Hick’s statement merely calling Jesus “one of the most and possibly the most wonderful human being who ever lived.”^39^ Albrow’s article was accompanied by a cartoon showing the crucified Jesus, but with a copy of *The Myth of God Incarnate* pinned at the top of the cross. Albrow was a Roman Catholic who had previously been the editor of *The Catholic Herald*.

*The Myth of God Incarnate* generated a swift response. As has been alluded to, four conservative Anglican and Roman Catholic theologians wrote a rebuttal volume titled *The Truth of God Incarnate* (which also prints John Macquarrie’s previously-published review of *The Myth of God Incarnate*).^40^ This book’s back cover identified the authors’ aim to refute “the damaging and misleading arguments” of the earlier book and asked, “*What is left of Christianity once a divine Christ is removed?*” The book’s preface uses cars as an analogy for Christianity and asks,

How much can you remove from a car, and still possess what is properly called a car? . . . We have seen many theologians engage so wholeheartedly in this process of rejecting some parts of the car and adapting others that we can scarcely believe their protestations that in reality the modernised version runs a lot better than the antiquated original.^41^

The preface adds that despite the fact *The Myth of God Incarnate* was written by people connected to the Church of England, its title sounds more like Communist propaganda.^42^ Most of the essays in *The Truth of God Incarnate* respond to specific arguments in *The Myth of God Incarnate*. The arguments in this rebuttal volume support the contention that the Church would lose its identity with the type of theology in *The Myth of God Incarnate*. The contributors were Michael Green (then the Rector of St. Aldate’s Church in Oxford and a former principal of St. John’s College, Nottingham), Christopher Butler (Auxiliary Bishop to the Archbishop of Westminster), Stephen Neill (a bishop and recently a faculty member at the University of Nairobi), Brian Hebblethwaite (Fellow and Dean of Chapel at Queens’ College, Cambridge), and Macquarrie (Lady Margaret Professor of Divinity at Oxford).^43^ Again, collectively, they represented a more theologically conservative section of the Church of England (although not Macquarrie to the same extent as the others). Green—the editor of *The Truth of God Incarnate*—notes that this rebuttal volume “was published within six weeks of its inception.”^44^ Despite this short window, Green found it humorous that *The Truth of God Incarnate* apparently “far outsold” *The Myth of God Incarnate*.^45^ Even so, he reflected later: “it seemed sad to me that it was necessary to use apologetics to controvert the writings of professed Christian scholars who, in fact, had allowed secularist presuppositions to cloud their Christian understanding.”^46^ The extent to which one agrees with the arguments in *The Truth of God Incarnate* will depend on the extent to which one agrees with its authors’ presuppositions, but the authors do a good job of showing where the earlier book lacks evidence for its claims. Still, the haste with which the book needed to be completed is unfortunate.

The debate did not end there. In 1978, George Carey wrote another response in the short book *God Incarnate: Meeting the Contemporary Challenges to a Classic Christian Doctrine*.^47^ At the time, Carey was the vicar of St. Nicholas Church in Durham, but he would later become the Archbishop of Canterbury in 1991. In addition, contributors to both *The Myth of God Incarnate* and *The Truth of God Incarnate* (among others) resumed their discussion in the 1979 publication *The Debate Continued: Incarnation and Myth*.^48^ This collection started as a series of papers that were shared at a gathering hosted by the University of Birmingham in July 1978. Most of the book is comprised of short papers from one side and then rebuttals from the other. In addition, although not *directly* referencing *The Myth of God Incarnate* and responding to a number of other things, Stephen W. Sykes’s book *The Integrity of Anglicanism* was likely influenced by *The Myth of God Incarnate*.^49^ Sykes’s book criticizes the Church of England of the late 1970s for losing its “integrity” because of its diversity of doctrinal beliefs; the book argues that such diversity threatens whether a unifying and coherent Anglican theology can even exist. In his preface, he seems to indirectly reference *The Myth of God Incarnate* controversy when he says: “Anglicans are never far from being painfully aware of their internal divisions, and at present these are vividly before the public.”^50^ Sykes’s critique is significant because he reminds the reader that a church must have shared essential truths to have “integrity.” He asks “whether or not Anglicanism has a coherent identity” and claims that “to inquire into the identity of Anglicanism is to ask whether there is any internal rationale binding Anglicans together as ‘church.’”^51^ Sykes’s book exists to *recover* a coherent identity for Anglicanism—an identity that seemingly was threatened by *The Myth of God Incarnate*. At the time he wrote it, Sykes was a professor at Durham University and he would eventually become the Bishop of Ely.

In addition, James D. G. Dunn’s book *Christology in the Making* (first published in 1980) “was partly prompted by the debate occasioned by *The Myth of God Incarnate*.”^52^ Although Dunn had briefly explored the Church’s Christology in his earlier book, *Unity and Diversity in the New Testament*, he recalls that “the publication of *The Myth of God Incarnate* and the controversy it aroused soon led me to the conclusion that a much fuller and more careful investigation of the whole area was called for.”^53^ In *Christology in the Making*, Dunn offers a thorough exploration of the development of the early church’s Christology and comes to less controversial conclusions than *The Myth of God Incarnate*, while also employing more rigorous scholarship.^54^ Although Dunn’s own views of Jesus were more orthodox than the authors of *The Myth of God Incarnate*, in contrast with the headline-grabbing aims of that publication, he wrote that his book was not going to attack or defend “any specific view of the incarnation,” but sought only to interpret what the New Testament authors meant.^55^ Dunn himself was a Methodist clergyman who taught at the University of Nottingham at the time and later moved to Durham University.

## The problem of audience

One of the most consistent and substantive criticisms of the book is that it was written in a way that would appeal neither to laypeople in churches nor serious academics. The pervasiveness of this criticism is remarkable and it is made even more compelling by the fact that it came from writers from a range of backgrounds. There are numerous examples of this in contemporary reviews. One person who made this argument was George Lindbeck—an American Lutheran who taught at Yale Divinity School. In Lindbeck’s review, he noted that “it contains much material of a specialized character” and asked whether it was for laypeople or academics.^56^ Lindbeck classified the book as “high-level popularization” but did not think it was as accessible as *Honest to God* or *The Secular City*.^57^ Edwards also found the book’s intended audience unclear, and wrote that the intended reader of the book

might be regarded as a mythological creature, for it would appear impossible that anyone capable of understanding this book has not already come face to face with the main problems of twentieth-century theology. Thus the book has much the same value as would be possessed by seven economists’ essays written in 1977 to suggest that we really must do something about inflation.^58^

Moreover, Edwards argues that the book does not do justice to recent theological scholarship on Christology and states, “the essayists write sometimes as if they had just discovered the questions—like a passenger discovering the Pacific from the window of a jet.”^59^ Similarly, Rodd compared *The Myth of God Incarnate* to John A. T. Robinson’s *Honest to God* but said that “it seems unlikely that it will have the impact of Bishop Robinson’s book since it is not written in the same popular style and it lacks the unity which a single writer can impose upon his work.”^60^ B. R. Brinkman—an English Jesuit—also found the book’s intended audience to be unclear and states that it “fails to envisage a reasonable homogeneity in its readership. Is the readership meant to be theologically perceptive or not? The title itself presumes a measure of theological gullibility.”^61^ Along a similar line of thought, the Roman Catholic New Testament scholar Luke Timothy Johnson begins his review by stating, “This book contains ten essays. Some have learning, some logic; few both.”^62^ Johnson describes Goulder’s essays as “puerile” from both a theological perspective and a historical one.^63^ Johnson also asks,

Are the authors writing for theologians, or a tractate for the masses? If for theologians, they offer nothing new and little competent . . . If the book is intended for popular consumption, it is somewhat dangerous. Not because the ideas advanced are essentially negative (though they are) and not because they are intellectually irresponsible (though they are, for the most part), but because they are accompanied by just enough specious learning to impress adolescents of all ages who persist in equating footnotes with authority.^64^

Reviewers attacked the book for being unintelligible to laypeople *and* academically substandard. Robinson observes the confusion in the book’s intended audience and claims that “the layman who has been persuaded to buy it is likely to get hopelessly bogged down. The publisher, if not the editor, ought to have decided on the market it was meant for and been much more severe.”^65^ He also describes Goulder’s contribution as “a triumph of ingenuity over judgment.”^66^ John F. Johnson criticizes the work’s lack of in-depth theological study and writes that

apart from a trivial reference to John of Damascus the book indicates not [sic] understanding of any theological work between the fifth and fifteenth centuries and not much after that until the nineteenth. Only such an omission could account for the uncritical view of God manifest there.^67^

Joanne McWilliam Dewart called the book unoriginal and wrote that the authors—and Wiles in particular—do not do justice to the history of patristic Christology; in her view, they put too much attention on the Alexandrian tradition and not enough on the Antiochene.^68^ George H. Tavard—an ordained Assumptionist Roman Catholic—offers another scathing review, concluding that

this volume is marred by its rationalist prejudice, and by its assumption that no previous period can possibly have understood the meaning of the NT. It is far below the level of scholarship that should be expected from the authors.^69^

In *Christianity Today*, John Stott wrote that “the book is unworthy of its highly competent contributors.”^70^ Stott was a conservative Anglican evangelical and formerly the longtime rector of All Souls Church, Langham Place, in London. R. K. Harrison of Wycliffe College, Toronto, wrote of the book: “If these writers are unwilling, or unable, to collect and assess all the relevant evidence in a fair and impartial manner without handling it selectively, they ought to turn their collective talents to something more beneficial to society.”^71^ Perhaps not surprisingly, Harrison has been described as “a staunch, if sometimes acerbic, defender of classical conservative opinions in Old Testament criticism.”^72^ Likewise, the Canadian institution he taught at—Wycliffe—is a strongly evangelical low-church Anglican seminary. Nonetheless, although many of these criticisms of *The Myth of God Incarnate* were written by theologically conservative Christians, even some reviewers who were sympathetic to the ideas in the book doubted it would achieve its aims. For example, the British Communist writer Philip Toynbee wrote of the book in *The Observer*:

what makes this a particularly odd manifesto is that much of it is written in a full blast of theological jargon, which will be quite unintelligible to everyone except fellow-theologians and a few *aficionados* like myself, so it can hardly be intended to make Christianity more palatable to the ordinary non-believer.^73^

Even though Toynbee *agreed* with the book’s view on Jesus, he also wrote that the book was unlikely to persuade “those who are deeply entrenched in orthodoxy” within the churches.^74^

The book’s reviewers made other criticisms. Edwards criticizes the authors for not recognizing the uniqueness of Jesus’s claims.^75^ Robinson believes that the book should have included an essay from a dogmatic or systematic theologian.^76^ Lindbeck asked a fundamental question about the book: “From where does one get, not the concepts for describing, but the norms for identifying God, for defining the divine, for evaluating religious experience?”^77^ This state of affairs may not be an accident. Given that Nineham contributed to the book, it is noteworthy that he had previously attacked the idea that any external “norms” (including certain ideas of the authority of Scripture) should regulate theology.^78^ Yet what is the alternative? Lindbeck observes that the essayists naively “adopt the old liberal assumption that enlightened reason and conscience have access to independent or transcendent criteria which enable them to pick and choose what is of highest value from within the various religious traditions.”^79^ Elsewhere, D. W. Walling—a scholar from North East London Polytechnic—criticized the authors for using a T. S. Eliot quote to support their perspective in the preface, when Eliot would not have endorsed the book’s rejection of Jesus’ divinity.^80^ Brinkman argues that the authors tried to cover too many issues and that “in practice the work assumes that there can be no views other than its own.”^81^ Furthermore, Brinkman claims that “the book fails to distinguish between proof and interpretation.”^82^ Willis observes that the contributors of the book seem to argue that “the fact that the doctrine of the incarnation was . . . culturally shaped is a reason for doubting its validity.”^83^ Willis argues that, on the contrary, “the cultural shaping of a particular doctrine and the multitude of cultural parallels are not a decisive argument against it.”^84^ Instead, Willis maintains that “the way the gospel . . . appropriated and transformed these cultural thought forms is a primary example of the truth and relevance of incarnational theology.”^85^ In short, reviewers found that *Myth* lacked sufficient backing for its core claims. In one way or another, these criticisms fit with the overarching perception that the work’s scholarship and argumentative rigor was lacking or incomplete, even though the book was simultaneously considered too difficult for a mass audience to understand. Furthermore, collectively, these reviews lead us to a common set of questions: what is ultimately the basis for the theological views expressed in the book? Are the arguments in the book only an extension of the authors’ Christological presuppositions, or do they have sufficient historical evidence? The former seems more likely than the latter.

Not all reviews condemned the book, although the positive assessments are a minority. A. J. Mattill Jr. preferred *The Myth of God Incarnate* to *The Truth of God Incarnate*, and noted that while the former might have destructive consequences, he did not think that was a bad thing, as Ecclesiastes 3:3 said there was “a time to break down and a time to build up.”^86^ There is a significant factor to keep in mind when assessing Mattill’s sympathetic review of the book: he was a Unitarian Universalist and the author of the 1979 book *A Christ for these Days: Toward a Unitarian Universalist Christology in the Twentieth Century*.^87^ Mattill was thus more predisposed to agree with the book than many Christian theologians. In addition, Ronald Quillo gives one of the most positive reviews of the book.^88^ Not too many other reviewers would likely agree with Quillo’s claim that “the book is hardly sensationalistic but is marked throughout by wise caution. Many counterviews are presented so fairly that they might well serve the authors’ opponents.”^89^ Quillo blames the controversy on the British press—not the book itself.^90^ Again, contrary to Quillo’s claims, it seems hard to believe that the authors of the book were not deliberately courting controversy by holding that press conference. Quillo taught at Spalding College, which was a Roman Catholic college in Kentucky. Regardless, for the most part, the book appeared unable to satisfy either lay readers or scholars.

## “The self-liquidation of the theological enterprise”

The Associated Press reported that the authors thought that it would “benefit the churches in an age of science if Jesus is regarded as a great teacher because many admire the wisdom of Jesus but cannot accept His supernatural aspects.”^91^ On the contrary, one cannot help but wonder why people would go to church at all if they accepted the book’s conclusions. As a parallel, in Ellis’s discussion of *Essays and Reviews*, Ellis writes that the authors of that book could not recognize that “the masses . . . would not be satisfied by doctrine merely shorn of its offensive intellectual items.”^92^ In the same way, it is unlikely that *The Myth of God Incarnate* inspired too many people to join the church. In effect, the book self-cannibalizes the church.

We can understand this point with reference to Berger’s work. Some of the statements on theology in Berger’s 1969 sociological study *A Rumor of Angels* describe *The Myth of God Incarnate* with remarkable accuracy.^93^ Hence, when discussing the radical theology of the era, Berger writes: “The self-liquidation of the theological enterprise is undertaken with an enthusiasm that verges on the bizarre.”^94^ Berger describes certain types of twentieth-century theology as follows:

The traditional religious affirmations are translated into terms appropriate to the new frame of reference, the one that allegedly conforms to the *Weltanschaaung* of modernity . . . The supernatural elements of the religious traditions are more or less completely liquidated . . . . The traditional lore, and in most cases the religious institution in charge of this lore as well, can then be presented as still or again “relevant” to modern man.^95^

Berger’s description almost perfectly fits the theological program of *The Myth of God Incarnate*. Indeed, McCabe wisely observed that most of the authors in *The Myth of God Incarnate* wanted “to commend Christianity to men (including themselves) who cannot believe in a ‘supernatural visitant’ and cannot believe that God has remained silent except in the Judeo-Christian tradition.”^96^ Similarly, when discussing Wiles’s essay in *The Myth of God Incarnate*, the British journalist George Gale said:

Wiles is very much in step with present university orthodoxy, which is so concerned to make Christianity acceptable to those who challenge its fundamental precepts that it is constantly throwing the baby out with the bath water, then slithering around on the bathroom floor trying to pick it up and put it back in again.^97^

C. Stephen Evans classified the authors of *Myth* as “adaptors,” which he defines as “those who cannot or do not wish to desert Christianity for some form of religious humanism or simply to become irreligious, but who cannot or do not wish to believe the substance of classical orthodox Christianity.”^98^ Yet Berger could observe the flaw in this sort of theology: the “benefits” of the new theology “are also available under strictly secular labels.”^99^ Specifically, Berger writes that “secularized Christianity” cannot always demonstrate “that the religious label, as modified in conformity with the spirit of the age, has anything special to offer.”^100^ Likewise, Berger argues that “for most people, symbols whose content has been hollowed out lack conviction or even interest. In other words, the theological surrender to the alleged demise of the supernatural defeats itself in precisely the measure of its success.”^101^ Berger concludes that this sort of theology destroys the institutions of the original theological tradition.^102^ This would be the outcome if the theology in *The Myth of God Incarnate* dominated the church.

Indeed, at the time, numerous responses to the book maintained that, if accepted, the ideas in *The Myth of God Incarnate* would destroy the church. A July 1977 Associated Press article noted: “Some critics say they fear the book will dismay Christians for whom Christ’s divinity is the core of their faith.”^103^ More pointedly, in *The Truth of God Incarnate*, John Macquarrie argues:

Christian doctrines are so closely interrelated that if you take away one, several others tend to collapse. After incarnation is thrown out, is the doctrine of the Trinity bound to go? What kind of doctrine of atonement remains possible? Would the Eucharist be reduced simply to a memorial service? . . . Finally, one has to ask whether such a reduced Christianity would move us either to acceptance or rejection. No doubt it would survive as literature, but hardly as a living religious faith.^104^

Similarly, Brian Hebblethwaite wrote that he felt he *must* refute the ideas in *The Myth of God Incarnate* and that those views are not “views which the church could ever endorse as permissible variants within the broad spectrum of its official doctrine.”^105^ Williams believed that the contributors to *The Myth of God Incarnate* offered “not only a devitalised theology . . . but a deracinated and immeasurably enfeebled religious life, which has no place for sacrament or contemplation, and precious little gospel for those condemned to die.”^106^ Anecdotally, Stott recalled that he was in Argentina when the book was published “and people were asking me if English churchmen were still Christians.”^107^ Evans also questions whether *The Myth of God Incarnate* leaves its readers with something that can still justifiably be called “Christianity.”^108^ Evans criticizes the authors of the book for claiming the term “Christian” for beliefs that fall outside what the faith has traditionally meant and adds that “in appropriating the term ‘Christian’ the adaptors are making a claim to be the legitimate heirs of this tradition and are supplanting the believers from their relationship to this tradition.”^109^ Evans finds it ironic that the “adaptors” claim to be using *logic* because they use historical criticism and “do not believe things which fly in the face of modern science”—but do not see a *logical problem* with using “language designed to exclude their position . . . to designate their position, regardless of the fact that this manoeuvre seems calculated to produce confusion.”^110^ It makes sense that Evans would want to maintain a clear and consistently orthodox definition of the word “Christian” because at the time he wrote this review, he taught at Wheaton College—an explicitly evangelical American liberal arts college. Elsewhere, like other critics, Willis questions whether the theology proposed in the book can still claim to be Christian in any meaningful sense when the authors seek to dispose of the doctrine of the Incarnation.^111^ Willis affirms that “the doctrine of the incarnation has been, and I would insist still is, a necessary, saving truth.”^112^ Although more sympathetic to the book than most, Malcolm L. Peel also noted the book’s lack of solutions; as Peel puts it: “though the authors have persuasively argued the case for the obsolescence of Chalcedonian Christology, they have done very little in terms of providing constructive alternatives.”^113^ Most reviewers, however, *did not* think the authors’ case against Chalcedonian Christianity was persuasive. McCabe notes with some irony that the book seems to reject Docetism (the idea that Jesus only *seemed* to be human) “because it is found to be incompatible with the European way of life in the second half of the twentieth century” and not because the Church has traditionally seen it as heretical.^114^ McCabe asserts that “what is peculiar to these authors is that they think the rejection of Docetism involves also the rejection of its contrary, the incarnation.”^115^ Alternatively, John Austin Baker chastises the authors for not taking the resurrection seriously—when it was “the affirmation from which the Christian faith and life began.”^116^ He concludes that by removing the resurrection from the picture, the authors “certainly made their task easier but they also made the finished product superficial and very much less helpful to the debate they desire than it might have been.”^117^ Baker himself was a canon at Westminster Abbey and later became the Bishop of Salisbury. In short, theological critics believed that the book discarded too many traditional doctrines to be recognizably Christian. Like other points noted in this article, this critique appears even stronger when one remembers that this position was expressed by Christians from a number of different theological traditions and by both Protestants and Catholics; it does not come from just one, narrowly-defined view of what Christianity is.

Adrian Hastings is another example of this phenomenon. Hastings critiqued the Church of England from the outside, as he was a Roman Catholic priest. Regardless, in his book *A History of English Christianity* (first published in 1985), Hastings writes that Anglican theological scholarship in the 1970s

was over-prone to appeal to an almost limitless pluralism as the only legitimate conclusion to draw from historical research in both biblical and ecclesiastical history, but it was far better at stressing the complexities of the evidence than at producing thereafter any sort of workable theological synthesis. This was in part because of the ever-increasing scepticism which the leading theologians of the English academic school—Dennis Nineham, Maurice Wiles, John Hick, Geoffrey Lampe and others—were evincing in regard to all the central dogmas most characteristic of Christianity, the incarnation, the Trinity, even for some the very existence of God.^118^

In other words, Hastings believed that the theological school associated with *The Myth of God Incarnate* offered a theology that was deconstructive but did not give real solutions. Hastings is scathing when he writes that *The Myth of God Incarnate* had “a rather over-confident note of debunking ‘The Incarnation.’”^119^ Hastings rightly recognized that “the consequence of their conclusions could hardly be other than the necessity of winding up historic Christianity . . . as unacceptable to the modern mind.”^120^ He adds that any excitement the book generated

was principally the smirking excitement of an agnostic world amused to witness the white flag hoisted so enthusiastically above the long-beleaguered citadel of Christian belief, the stunning excitement of the rank and file of weary defenders on learning that their staff officers had so light-heartedly ratted on them.^121^

Hastings was not the only one to criticize the book’s smug tone. As Gill put it,

the authors tend to strike a condescending note when speaking of the theological beliefs and/or language of earlier Christian thinkers. Repeatedly we are told, in effect, that those incarnational modes of thought may have served more primitive minds well, but they will hardly do for us today. This might be termed the “man come of age fallacy”—the assumption that later interpretations are ipso facto better, and that at any cost the contemporary mind must not be offended.^122^

Likewise, comparing these Anglican scholars to those in past generations, Hastings concludes that “the theology of Gore, Temple, Ramsey or Farrer was, most certainly, one the Church could live and thrive with. The same cannot be said for that of Nineham, Hick, or Cupitt.”^123^ He rightly argues that “there is simply no future for a Church which can produce no reasoned expression of its faith stronger than what the dominant theologians of the seventies were able to muster.”^124^

To that end, the ecclesiastical status of the authors undoubtedly intensified the controversy. Ellis writes of *Essays and Reviews* that “had the essays been written by laymen, little would have been heard of them, but they seemed to be a *trahison des clercs* which must weaken the hold on one’s congregation.”^125^ The position of *The Myth of God Incarnate* is similar. For people in the pews, if these opinions were held by leading figures in the Church, where could they turn? Thus, others *within* the Church of England went to great lengths to repudiate the book.

To put an exclamation mark on things, some of the contributors to *The Myth of God Incarnate* simply stopped believing in God altogether after the book was published.^126^ In 1981—four years after *The Myth of God Incarnate*—Cupitt explained why he rejected traditional belief in God in his book *Taking Leave of God*.^127^ Likewise, another contributor to *The Myth of God Incarnate*—Michael Goulder—resigned from the Church and explained why he no longer believed in the 1983 book *Why Believe in God?*^128^ Goulder co-authored that book with fellow *Myth of God Incarnate* contributor John Hick. Hick at least believed in God, but in a more pluralistic sense that presented the world’s major religions as essentially equal.^129^ Nonetheless, if followed to its logical end, the book’s theology would lead someone outside the church—as ultimately happened to some of its own authors.

## Conclusions

Biblical and theological scholarship since the 1980s has not supported the book’s conclusions.^130^ Given that Nineham contributed to the book, it is telling that Hastings contrasts Nineham’s 1963 Mark commentary with Martin Hengel’s 1985 volume *Studies in the Gospel of Mark* as evidence of “the very considerable reversal of seemingly assured anti-conservative positions by the 1980s.”^131^ Hick’s former doctoral student William Lane Craig—ironically, a conservative evangelical—observes that since *The Myth of God Incarnate* was published, New Testament scholars (including those who are atheists) generally have had more optimism in their ability to use the Gospels as historical sources.^132^ It is also significant that since the 1980s, New Testament scholarship has put more emphasis on the *Jewishness* of Jesus and the New Testament.^133^ E. P. Sanders’s 1985 book *Jesus and Judaism* and John P. Meier’s *A Marginal Jew* were important and representative works in this regard.^134^ In contrast, *The Myth of God Incarnate* put too much emphasis on Hellenistic influence on Christianity.

Ultimately, the book’s reception is tied to a few main points. It did not have a clear target because academic reviewers found it to be substandard scholarship, while not being easy to read for lay Christians either. Rather, it seems inevitable that it would just offend the latter. *The Myth of God Incarnate* worked neither as a popular nor a serious academic book. For that matter, the reasons the book was offensive to many Christians leads into our next point. As we have seen following Berger’s example, despite being written by people who were (at that time at least) supposed to be leaders in the church, the book argued a case that—despite its authors’ claims—would undercut the church’s reason for existence if accepted. Although he was not discussing *The Myth of God Incarnate* specifically, Scot McKnight has observed that “the church is rooted in a Christology and the change of the latter entails changing the former.”^135^ McKnight reflects that it would be wrong for the church “to re-do its Christology every generation.”^136^ McKnight imagines a church denomination tabling a vote about a revised Christology at a meeting and finds such a scene to be absurd.^137^ A book like *The Myth of God Incarnate* was thus inevitably going to create division in the Church of England. Furthermore, as Berger said, “secularized Christianity” struggles to justify its own distinct existence after a certain point.^138^ The challenge for the church today is to determine how to harmonize its beliefs with biological, scientific, and historical knowledge without losing its identity.

In the end, however, it is undeniable that—in spite of the book’s many flaws—the publication of *The Myth of God Incarnate* was nonetheless a watershed in late twentieth-century English Anglican theology and one that is directly tied to how the next generation of Anglican theologians responded to the book and what methods they used. In other words, the book created a proverbial fork in the road for the next generation of Anglican theologians to determine their way forward. For example, while lecturing at the University of Birmingham from 1976 to 1991, David Ford moved his theology away from the patristic study and historical-critical biblical scholarship that had previously dominated Anglican theology. Instead, particularly in his 1981 book *Barth and God’s Story*, Ford chose to move in a Barthian direction.^139^ Then, the Scottish Anglican scholar David Brown tried a different approach. In his 1985 book *The Divine Trinity*, Brown attempted to critique *The Myth of God Incarnate* on grounds that might be more persuasive to the book’s original authors.^140^ Thus, in the first chapter of *The Divine Trinity*, Brown references *The Myth of God Incarnate* but then says,

The subsequent popular reply, *The Truth of God Incarnate*, fell like a damp squib simply because the presuppositions of the contributors were so different from those held by contributors to the other volume, and yet no serious attempt was made to challenge that alternative perspective at its most fundamental roots; instead, familiar arguments were once again rehearsed, arguments that had already been rejected by their opponents because of these unexamined fundamental roots. Nor does it seem to me that the situation was much improved by later more academic contributions to the debate . . . For, while there was undoubtedly some widening of the discussion, it was a process that was not carried nearly far enough.^141^

In the rest of this book, Brown discusses traditional theological concepts such as “Divine Action,” the Incarnation, the Holy Spirit, and (especially) Trinitarian theology, but tries to be fair to Goulder, Wiles, and other contributors to *The Myth of God Incarnate*. For example, he agrees with Goulder “that a high consciousness in Jesus cannot of itself guarantee Christ’s divinity,” but comes to somewhat different conclusions.^142^ He also cautiously accepts some of Frances Young’s statements on Christology in *The Myth of God Incarnate*.^143^ Brown notes that the position of *The Myth of God Incarnate* involves a “thoroughly non-interventionalist” portrayal of God, “with God set very much at a distance from the world.”^144^ Even so, Brown believes that while this view of God has some logical coherency, he nonetheless defends an interventionist view of God.^145^ In addition, Brown holds that philosophical theologians can correct the excesses of some biblical scholars by helping them avoid “the false equation of historical original with theological truth.”^146^ Brown maintains that New Testament scholars sometimes bring faulty assumptions to their work because “all historians try to get back, so far as possible, to what one might call the historical original, what actually happened or was said,” but “this carries with it the danger of inferring that that is all that can or need be said on the matter; in short, that the historical original is none other than what is ultimately or theologically true.”^147^ Brown believes that philosophical theology can address this issue, and, incidentally, this limitation is characteristic of *The Myth of God Incarnate*.

Overall, Brown’s book is an intellectually impressive achievement, but not many subsequent Anglican theologians have followed his lead. It should not go unnoticed that—compared with Brown—a book such as *The Truth of God Incarnate* provides a position that is much more immediately comprehensible for lay Christians. The subtleties of Brown’s book may appeal to academic theologians, but are less transferable to the Church at large. Brown himself acknowledged that “the likely readership” for his book would “consist mostly of theologians,” albeit those with limited philosophical knowledge to begin with.^148^ Like *The Myth of God Incarnate* itself, Brown’s book is not particularly accessible to lay Christians. Even so, given that *The Myth of God Incarnate* was—even in a flawed way—pitched at the masses means that attempts to engage with the book should be readable among the masses to have their full effect. *The Truth of God Incarnate* succeeds by that standard. While Brown may have hoped to make arguments that would be persuasive to the authors of *The Myth of God Incarnate*, one suspects that the contributors to *The Truth of God Incarnate* were more concerned with defending their positions to lay Christians whose faith may have been threatened, rather than addressing the authors of the other book on their own ground. In any event, the fact that nearly five decades have passed has now lessened the place of *The Myth of God Incarnate* in the broader Anglican consciousness.

If nothing else, even if its conclusions had been better grounded, *The Myth of God Incarnate* exemplifies how *not* to communicate radical or newer concepts in theology to the masses. Albert Schweitzer once wisely noted “that in theology the most revolutionary ideas are swallowed quite readily so long as they smooth their passage by a few small concessions” but not “when a spicule of bone stands out obstinately and causes choking.”^149^ Not only were the ideas in *The Myth of God Incarnate* hard for many Christians to swallow, but they were presented in a way that made choking inevitable.

## Footnotes

1 John Hick, ed. *The Myth of God Incarnate* (London: SCM Press, 1977).

2 Ieuan Ellis, *Seven Against Christ: A Study of ‘Essays and Reviews,’* Studies in the History of Christian Thought 23 (Leiden: E. J. Brill, 1980).

3 Ibid., 155.

4 Michael Goulder, foreword to *The Debate Continued: Incarnation and Myth*, ed. Michael Goulder (Grand Rapids, MI: William B. Eerdmans, 1979), vii.

5 Peter L. Berger, *A Rumor of Angels: Modern Society and the Rediscovery of the Supernatural* (Garden City, N.Y.: Doubleday, 1969), 14.

6 John Hick, preface to *The Myth of God Incarnate*, ix–x.

7 Maurice Wiles, “Christianity without Incarnation?” in *The Myth of God Incarnate*, 9.

8 Frances Young, “A Cloud of Witnesses,” in *The Myth of God Incarnate*, 42.

9 Michael Goulder, “Jesus, the Man of Universal Destiny,” in *The Myth of God Incarnate*, 48–63; Michael Goulder, “The Two Roots of the Christian Myth,” in *The Myth of God Incarnate*, 64–86.

10 Frances Young, “Two Roots or a Tangled Mess?” in *The Myth of God Incarnate*, 87–121.

11 Maurice Wiles, “Myth in Theology,” in *The Myth of God Incarnate*, 148–66.

12 John Hick, “Jesus and the World Religions,” in *The Myth of God Incarnate*, 167–85.

13 F. F. Bruce, *In Retrospect: Remembrances of Things Past* (Grand Rapids, MI: William B. Eerdmans, 1980), 227 n. 3.

14 Ibid.

15 David L. Edwards, “From the Archive: David L. Edwards Reviews *The Myth of God Incarnate*,” review of *The Myth of God Incarnate*, ed. by John Hick, *Church Times*, April 30, 2018. Accessed December 3, 2020. https://www.churchtimes.co.uk/articles/2018/4-may/comment/opinion/from-the-archive-david-l-edwards-reviews-the-myth-of-god-incarnate. First published on July 1, 1977.

16 Ibid.

17 For an obituary of Edwards, see “Obituary: The Very Revd Dr David L. Edwards,” *Church Times*, May 4, 2018, Accessed December 9, 2024. https://www.churchtimes.co.uk/articles/2018/4-may/gazette/obituaries/obituary-the-very-revd-dr-david-l-edwards.

18 Tom Harpur, “Group of U. K. Scholars Questions Christ’s Divinity: New Book by Seven British Theologians Expected to Stir Religious Controversy,” *The Toronto Star*, July 9, 1977, 13.

19 Ibid.

20 John F. Johnson, “God Incarnate: Myth or Truth?,” review of *The Myth of God Incarnate*, ed. John Hick, *Presbyterion* 4, no. 2 (1978): 95.

21 John A. T. Robinson, “The Myth of God Incarnate—The Truth of God Incarnate,” review of *The Myth of God Incarnate*, ed. John Hick, and *The Truth of God Incarnate*, ed. Michael Green, *Journal of Theology for Southern Africa* 21, no. 1 (1977): 68.

22 Ibid.

23 John A. T. Robinson, *Honest to God* (London: SCM Press, 1963).

24 John A. T. Robinson, “The Debate Continues,” in *The Honest to God Debate*, ed. by David L. Edwards (London: SCM Press, 1963), 234–35. For the original article, see John Robinson, “Our Image of God Must Go,” *The Observer*, March 17, 1963, 21.

25 Robinson, “The Debate Continues,” 235.

26 Herbert McCabe, “The Myth of God Incarnate,” review of *The Myth of God Incarnate*, ed. John Hick, *New Blackfriars* 58 (no. 687): 350.

27 David Willis, review of *The Myth of God Incarnate*, ed. John Hick, *Theology Today* 35, no. 2 (1978): 222.

28 Rowan Williams, “Myth and Faith: Some Reflections on ‘The Myth of God Incarnate,’” *Theoria to Theory* 11, no. 3 (1977): 203–12.

29 Ibid., 212.

30 John A. T. Robinson, *Truth is Two-Eyed* (London: SCM Press, 1979), 120.

31 Robinson, “The Myth of God Incarnate,” 70.

32 Alasdair Heron, “Doing Without the Incarnation?,” review of *The Myth of God Incarnate*, ed. John Hick, and *The Truth of God Incarnate*, ed. Michael Green, *Scottish Journal of Theology* 31, no. 1 (1978): 70–71.

33 Johnson, “God Incarnate,” 100.

34 James E. Griffiss, review of *Incarnation and Myth: The Debate Continued*, ed. Michael Goulder, and *The Myth/Truth of God Incarnate*, ed. Durstan R. McDonald, *Saint Luke’s Journal of Theology* 24, no. 4 (1981): 307.

35 Jerry H. Gill, “Myth and Incarnation,” review of *The Myth of God Incarnate*, ed. John Hick, *The Christian Century*, December 21, 1977, 1190; John Stott, “Is the Incarnation a Myth?,” review of *The Myth of God Incarnate*, ed. John Hick, *Christianity Today*, November 4, 1977, 34.

36 Cyril S. Rodd, “Talking Points From Books,” review of *The Myth of God Incarnate*, ed. John Hick, *The Expository Times* 89, no. 1 (1977): 4.

37 Ibid.

38 Desmond Albrow, “Have Pity on Us Poor Christians,” *Sunday Telegraph*, July 3, 1977, 18.

39 Ibid.

40 Michael Green, ed., *The Truth of God Incarnate* (London: Hodder & Stoughton, 1977).

41 Preface to *The Truth of God Incarnate*, ed. Michael Green (London: Hodder & Stoughton, 1977), 9.

42 Ibid., 13.

43 See “Publisher’s Acknowledgments,” in *The Truth of God Incarnate*, 5.

44 Michael Green, *Adventure of Faith: Reflections on Fifty Years of Christian Service*(Grand Rapids, MI: Zondervan, 2001), 141.

45 Ibid.

46 Ibid.

47 George Carey, *God Incarnate: Meeting the Contemporary Challenges to a Classic Christian Doctrine* (Westmont, IL: InterVarsity Press, 1978).

48 Michael Goulder, ed., *The Debate Continued: Incarnation and Myth* (Grand Rapids, MI: William B. Eerdmans, 1979).

49 Stephen W. Sykes, *The Integrity of Anglicanism* (Oxford: A.R. Mowbray & Co, 1979).

50 Ibid., ix.

51 Ibid., 1.

52 James D. G. Dunn, “In Quest of Truth,” in *I (Still) Believe: Leading Bible Scholars Share Their Stories of Faith and Scholarship*, ed. John Byron and Joel N. Lohr (Grand Rapids, MI: Zondervan, 2015), 61. See James D. G. Dunn, *Christology in the Making: A New Testament Inquiry into the Origins of the Doctrine of the Incarnation*, 2nd ed. (Grand Rapids, MI: William B. Eerdmans, 1989 [1980]).

53 Dunn, *Christology in the Making*, 6. Cf. James D. G. Dunn, *Unity and Diversity in the New Testament: An Inquiry into the Character of Earliest Christianity*, 2nd ed. (London: SCM Press, 1990 [1977]).

54 Dunn, *Christology in the Making*.

55 Ibid., 10.

56 George Lindbeck, review of *The Myth of God Incarnate*, ed. John Hick, *The Journal of Religion* 59, no. 2 (1979): 248.

57 Ibid.

58 Edwards, “From the Archive.”

59 Ibid.

60 Rodd, “Talking Points From Books,” 1.

61 B. R. Brinkman, review of *The Origin of Christology*, by C. F. D. Moule, and *The Myth of God Incarnate*, ed. John Hick, *Heythrop Journal* 19, no. 1 (1978): 73.

62 Luke T. Johnson, review of *The Myth of God Incarnate*, ed. John Hick, *Religious Education: The Official Journal of the Religious Education Association* 73, no. 2 (1978): 252.

63 Ibid., 252.

64 Ibid., 252–54. There does not seem to be a page 253.

65 Robinson, “The Myth of God Incarnate,” 67.

66 Ibid., 67.

67 Johnson, “God Incarnate,” 100.

68 Joanne McWilliam Dewart, “Christological Particularity: Need It Be a Scandal?” *Anglican Theological Review* 62, no. 1 (1980): 64–74.

69 George H. Tavard, review of *The Myth of God Incarnate*, ed. John Hick, *Theological Studies* 39, no. 1 (1978): 166.

70 Stott, “Is the Incarnation a Myth,” 34.

71 R. K. Harrison, “Professor Says Old Argument Is Based on Flimsy Evidence,” *The Toronto Star*, July 9, 1977, 13.

72 Gerald Bray, *Biblical Interpretation: Past & Present* (Downer’s Grove, IL: IVP Academic, 1996), 547.

73 Philip Toynbee, “How Divine Was Jesus?” *The Observer*, July 3, 1977, 10.

74 Ibid.

75 Edwards, “From the Archive.”

76 Robinson, “The Myth of God Incarnate,” 69.

77 Lindbeck, review of *The Myth of God Incarnate*, 249.

78 D. E. Nineham, “The Use of the Bible in Modern Theology,” *Bulletin of the John Rylands Library* 52 (no. 1) 1969: 178–99; D. E. Nineham, “Wherein Lies the Authority of the Bible,” in *On the Authority of the Bible*, ed. Leonard Hodgson (London: SPCK, 1960), 81–96.

79 Lindbeck, review of *The Myth of God Incarnate*, 249.

80 D. W. Walling, “T. S. Eliot and ‘The Myth of God Incarnate,’” *Expository Times* 91, no. 1 (1979): 22. Cf. Hick, preface to *The Myth of God Incarnate*, ix.

81 Brinkman, review of *The Origin of Christology*, by C. F. D. Moule, and *The Myth of God Incarnate*, ed. John Hick, 73.

82 Ibid.

83 Willis, review of *The Myth of God Incarnate*, 221.

84 Ibid., 222.

85 Ibid.

86 A. J. Mattill Jr., “God Incarnate: Myth or Truth?,” review of *The Myth of God Incarnate*, ed. John Hick, and *The Truth of God Incarnate*, ed. Michael Green, *Perspectives in Religious Studies* 7, no. 3 (1980): 236.

87 A. J. Mattill Jr., *A Christ for these Days: Toward a Unitarian Universalist Christology in the Twentieth Century* (Boston: Church of the Larger Fellowship, 1979).

88 Ronald Quillo, review of *The Myth of God Incarnate*, ed. John Hick, *Horizons* 5, no. 2 (1978): 269–70.

89 Ibid., 269.

90 Ibid.

91 Associated Press, “7 Scholars Challenge Divinity of Christ,” *The Charlotte Observer*, July 1, 1977, 1.

92 Ellis, *Seven Against Christ*, 97.

93 See Berger, *A Rumor of Angels*.

94 Ibid., 14.

95 Ibid., 25.

96 McCabe, “The Myth of God Incarnate,” 356.

97 Quoted in “‘A Shilling Shocker’ Says Church Times,” *The Toronto Star*, July 9, 1977, 13.

98 C. Stephen Evans, “Mis-using Religious Language: Something About Kierkegaard and ‘The Myth of God Incarnate,” *Religious Studies* 15, no. 2 (1979): 139.

99 Berger, *A Rumor of Angels*, 25.

100 Ibid., 25–26.

101 Ibid., 26.

102 Ibid.

103 “‘A Shilling Shocker’ Says Church Times.”

104 John Macquarrie, “Postscript: Christianity without Incarnation? Some Critical Comments,” in *The Truth of God Incarnate*, 144.

105 Brian Hebblethwaite, “*The Myth* and Christian Faith,” in *The Debate Continued*, 16.

106 Williams, “Myth and Faith,” 212.

107 Stott, “Is the Incarnation a Myth?” 34.

108 Evans, “Mis-using Religious Language,” 148–50.

109 Ibid., 149–50.

110 Ibid., 150.

111 Willis, review of *The Myth of God Incarnate*, 222.

112 Ibid.

113 Malcolm L. Peel, “Christology in a New Mode,” review of *The Myth of God Incarnate*, ed. John Hick, *The Christian Century*, December 7, 1977, 1147.

114 McCabe, “The Myth of God Incarnate,” 350.

115 Ibid.

116 John Austin Baker, review of *The Myth of God Incarnate*, ed. John Hick, *The Journal of Theological Studies* 29, no. 1 (1978): 297.

117 Ibid.

118 Adrian Hastings, *A History of English Christianity, 1920–2000*, 4th ed. (London: SCM Press, 2001), 649.

119 Ibid., 650.

120 Ibid.

121 Ibid.

122 Gill, “Myth and Incarnation,” 1190.

123 Hastings, *A History of English Christianity*, 663.

124 Ibid.

125 Ellis, *Seven against Christ*, 203.

126 Cf. Hastings, *A History of English Christianity*, 650–51.

127 Don Cupitt, *Taking Leave of God* (London: SCM Press, 1981).

128 Michael Goulder and John Hick, *Why Believe in God* (London: SCM Press, 1983).

129 Cf. John Hick, *God and the Universe of Faiths: Essays in the Philosophy of Religion* (London: Macmillan, 1973).

130 Cf. Hastings, *A History of English Christianity*, 653.

131 Ibid., 703 n. 4. See D. E. Nineham, *The Gospel of St Mark*, The Pelican Gospel Commentaries (Baltimore: Penguin Books, 1963); Martin Hengel, *Studies in the Gospel of Mark* (Philadelphia: Fortress Press, 1985).

132 William Lane Craig, *On Guard: Defending Your Faith With Reason and Precision* (Colorado Springs, CO: David C. Cook, 2010), 183; 196.

133 See Stephen Neill and Tom Wright, *The Interpretation of the New Testament: 1861–1986*, 2nd ed. (Oxford: Oxford University Press, 1988), 397–98; Helen K. Bond, *The Historical Jesus: A Guide for the Perplexed* (London and New York: T & T Clark, 2012), 20.

134 E. P. Sanders, *Jesus and Judaism* (London: SCM Press, 1985); John P. Meier, *A Marginal Jew: Rethinking the Historical Jesus*, vol. 1, *The Roots of the Problem and the Person* (New York: Bantam Doubleday, 1991).

135 Scot McKnight, “Why the Authentic Jesus Is of No Use to the Church,” in *Jesus, Criteria, and the Demise of Authenticity*, ed. Chris Keith and Anthony Le Donne (New York: T&T Clark, 2012), 185.

136 Ibid., 184.

137 Ibid.

138 Berger, *A Rumor of Angels*, 25–26.

139 David Ford, *Barth and God’s Story: Biblical Narrative and the Theological Method of Karl Barth in the “Church Dogmatics”* (Frankfurt am Main: Lang, 1981).

140 David Brown, *The Divine Trinity* (La Salle, IL: Open Court Publishing Company, 1985).

141 Ibid., 3.

142 Ibid., 108.

143 Ibid., 147.

144 Ibid., 239.

145 Ibid.

146 Ibid., 103.

147 Ibid., 103–104.

148 Ibid., 220.

149 Albert Schweitzer, *The Quest of the Historical Jesus*, first completed edition, Fortress Classics in Biblical Studies, trans. W. Montgomery, J. R. Coates, Susan Cupitt, and John Bowden, ed. John Bowden (Minneapolis: Fortress Press, 2001), 35–36.

